# Supplementary material for: Clinical and imagenologic significance of the neutrophil-to-lymphocyte ratio in neuromyelitis optica spectrum disorder: A systematic review with meta-analysis
Source: PLoS One. 2023 Feb 9;18(2):e0281064. doi: 10.1371/journal.pone.0281064 (PMC9910629; doi:10.1371/journal.pone.0281064)
Supplement: S1 Table — (DOCX) [file pone.0281064.s002.docx]

**S1 Table. Search strategy**

| Database | Search strategy | Results |
| --- | --- | --- |
| Pubmed | #1 Neuromyelitis Optica [MH] OR “Neuromyelitis Optic*”[all fields] OR NMOSD*[all fields] OR Devic's[all fields] OR Devics[all fields] OR Devic[all fields]  #2 "neutrophil to lymphoc*" [all fields] OR "neutrophil-to-lymphoc*"[all fields] OR "neutrophil/lymphoc*"[all fields] OR "NLR" [all fields] OR "neutrophil lymphoc*" [all fields] OR "granulocyte lymphoc*" [all fields] OR "granulocyte to lymphoc*" [all fields] OR "granulocyteto-lymphoc*"[all fields] OR "granulocyte/lymphoc*" OR "GLR" [all fields]  #3: #1 AND #2 | 10 |
| Scopus | #1 ALL (“Neuromyelitis Optic*” OR NMO* OR Devic's OR Devics OR Devic)  #2 ALL ("neutrophil to lymphoc*" OR “neutrophil-to-lymphoc*” OR “neutrophil/lymphoc*” OR "NLR" OR "neutrophil lymphoc*" OR "granulocyte lymphoc*" OR “granulocyte to lymphoc*” OR “granulocyteto-lymphoc*” OR “granulocyte/lymphoc*” OR "GLR")  #3: #1 AND #2 | 187 |
| WOS | #1 TS= (“Neuromyelitis Optic*” OR NMO* OR Devic's OR Devics OR Devic) OR TI= (“Neuromyelitis Optic*” OR NMO* OR Devic's OR Devics OR Devic) OR AB= (“Neuromyelitis Optic*” OR NMO* OR Devic's OR Devics OR Devic) OR AK= (“Neuromyelitis Optic*” OR NMO* OR Devic's OR Devics OR Devic) OR KP= (“Neuromyelitis Optic*” OR NMO* OR Devic's OR Devics OR Devic)  #2 TS=("neutrophil to lymphoc*" OR “neutrophil-to-lymphoc*” OR “neutrophil/lymphoc*” OR "NLR" OR "neutrophil lymphoc*" OR "granulocyte lymphoc*" OR “granulocyte to lymphoc*” OR “granulocyteto-lymphoc*” OR “granulocyte/lymphoc*” OR "GLR") OR TI=("neutrophil to lymphoc*" OR “neutrophil-to-lymphoc*” OR “neutrophil/lymphoc*” OR "NLR" OR "neutrophil lymphoc*" OR "granulocyte lymphoc*" OR “granulocyte to lymphoc*” OR “granulocytetolymphoc*” OR “granulocyte/lymphoc*” OR "GLR") OR AB=("neutrophil to lymphoc*" OR “neutrophil-to-lymphoc*” OR “neutrophil/lymphoc*” OR "NLR" OR "neutrophil lymphoc*" OR "granulocyte lymphoc*" OR “granulocyte to lymphoc*” OR “granulocyteto-lymphoc*” OR “granulocyte/lymphoc*” OR "GLR") OR AK=("neutrophil to lymphoc*" OR “neutrophil-tolymphoc*” OR “neutrophil/lymphoc*” OR "NLR" OR "neutrophil lymphoc*" OR "granulocyte lymphoc*" OR “granulocyte to lymphoc*” OR “granulocyteto-lymphoc*” OR “granulocyte/lymphoc*” OR "GLR") OR KP=("neutrophil to lymphoc*" OR “neutrophil-tolymphoc*” OR “neutrophil/lymphoc*” OR "NLR" OR "neutrophil lymphoc*" OR "granulocyte lymphoc*" OR “granulocyte to lymphoc*” OR “granulocyteto-lymphoc*” OR “granulocyte/lymphoc*” OR "GLR")  #3: #1 AND #2 | 40 |
| Google Scholar | #1 (neuromyelitis optica OR nmosd)  #2 ('neutrophil lymphocyte ratio' OR nlr)  #3: #1 AND #2 | 100 |
| Embase | #1 ('myelooptic neuropathy'/exp OR 'devic disease' OR 'devic syndrome' OR 'myelooptic neuropathy' OR 'myeloopticoneuropathy' OR 'myeloptico neuropathy' OR 'myelopticoneuropathy' OR 'neuromyelitis optica' OR 'neuromyelitis optica spectrum disorder' OR 'neuropticomyelitis' OR 'optic neuromyelitis' OR nmosd)  #2 ('neutrophil lymphocyte ratio'/exp OR 'nlr (lymphocyte)' OR 'neutrophil lymphocyte ratio' OR 'neutrophil to lymphocyte ratio' OR 'neutrophil/lymphocyte ratio')  #3: #1 AND #2 | 39 |
