# Supplementary material for: Clinical and imagenologic significance of the neutrophil-to-lymphocyte ratio in neuromyelitis optica spectrum disorder: A systematic review with meta-analysis
Source: PLoS One. 2023 Feb 9;18(2):e0281064. doi: 10.1371/journal.pone.0281064 (PMC9910629; doi:10.1371/journal.pone.0281064)
Supplement: S2 Table — (DOCX) [file pone.0281064.s003.docx]

**S2 Table. Excluded studies**

| Author-year | Title | Exclusion reasons |
| --- | --- | --- |
| Aubé (2014) | Neutrophils mediate blood–spinal cord barrier disruption in demyelinating neuroinflammatory diseases | No population |
| Benetou (2020) | Neutrophil-to-lymphocyte ratio correlates with disease activity in myelin oligodendrocyte glycoprotein antibody associated disease (MOGAD) in children | No population |
| Berti (2019) | Neutrophil-to-lymphocyte ratio as a biomarker to differentiate paediatric MOG+ recurrent demyelinating syndrome from multiple sclerosis | Conference Abstract |
| Carnero (2020) | The neutrophil-to-lymphocyte ratio in aquaporin-4-positive nmosd patients: a latin american multicenter study | Conference Abstract |
| Carnero (2021) | Platelet-to-lymphocyte ratio as a peripheral biomarker that may help to differentiate MS from NMOSD at disease onset | Conference Abstract |
| Guclu (2015) | Elevated neutrophil lymphocyte ratio in recurrent optic neuritis | Other outcome |
| Nayanajeehwi (2020) | Multiple sclerosis and neuromyelitis optica spectrum disorders: Comparison of oxidative burden and Vitamin D level | Conference Abstract |
| Peternell (2021) | Blood parameter analysis in pediatric MOG-antibodyassociated disorders | Conference Abstract |
| Sun (2021) | Investigation of the Neutrophil-to-lymphocyte Ratio and Platelet-  to-lymphocyte Ratio in Patients with Neuromyelitis Optica Spectrum Disorders | Pre-print |
| Tong (2021) | Serum Immunoglobulin G Level and Neutrophils to Lymphocytes Ratio Associated with the Prognosis of Neuromyelitis Optica Spectrum Disorder | Full text no available |
| Yu (2017) | The correlation between cerebrospinal fluid protein and disability status in patients with neuromyelitis optica spectrum disorders | Full text no available |
