# Supplementary material for: Clinical and imagenologic significance of the neutrophil-to-lymphocyte ratio in neuromyelitis optica spectrum disorder: A systematic review with meta-analysis
Source: PLoS One. 2023 Feb 9;18(2):e0281064. doi: 10.1371/journal.pone.0281064 (PMC9910629; doi:10.1371/journal.pone.0281064)
Supplement: S3 Table — (DOCX) [file pone.0281064.s004.docx]

**S3 Table: NEWCASTLE - OTTAWA QUALITY ASSESSMENT SCALE FOR INCLUDED STUDIES**

| ***NEWCASTLE - OTTAWA QUALITY ASSESSMENT SCALE FOR COHORT STUDIES*** | | | | | | | | | | | |
| --- | --- | --- | --- | --- | --- | --- | --- | --- | --- | --- | --- |
| ***STUDY*** | **SELECTION** | | | | **COMPARABILITY** | **OUTCOME** | | |  |  |  |
|  | **Representativeness of the exposed cohort** | **Sample size** | **Ascertainment of exposure** | **Definition of cohorts** | **Comparability of Cohorts on the Basis of the Design or Analysis Maximum: ☆☆** | **Assessment of outcome** | **Statistical test** | **Adequacy of follow up of cohorts** | **SCORE** | **Risk of bias** |  |
| *Carnero et al* |  |  | ☆ | ☆ | ☆☆ | ☆ | ☆ |  | *6* | Low risk of bias |  |
| *Chen et al* | ☆ |  | ☆ | ☆ | ☆☆ | ☆ | ☆ |  | *7* | Low risk of bias |  |
| *Lin et.al* | ☆ |  | ☆ | ☆ |  | ☆ | ☆ |  | *5* | High Risk of bias |  |
| *Xie et.al* | ☆ |  | ☆ | ☆ | ☆☆ | ☆ | ☆ |  | *7* | Low Risk of bias |  |
| *Zhou et.al* | ☆ |  | ☆ | ☆ | ☆☆ | ☆ | ☆ |  | *7* | Low Risk of bias |  |
| *Yangyang et al* | ☆ |  | ☆ | ☆ | ☆☆ | ☆ | ☆ |  | *7* | Low Risk of bias |  |
