# Supplementary material for: Clinical and imagenologic significance of the neutrophil-to-lymphocyte ratio in neuromyelitis optica spectrum disorder: A systematic review with meta-analysis
Source: PLoS One. 2023 Feb 9;18(2):e0281064. doi: 10.1371/journal.pone.0281064 (PMC9910629; doi:10.1371/journal.pone.0281064)
Supplement: Attachment — Submitted filename: RESPONSE TO REVIEWERS.docx [file pone.0281064.s005.docx]

**RESPONSE TO REVIEWERS**

1. **Is the manuscript technically sound and do the data support the conclusions?**

The manuscript was rewritten with the editors' corrections.

2. **Has the statistical analysis been adequately and rigorously performed?**

The statistical analysis was reevaluated in the results according to the editors' comments.

3. **Have the authors provided all the data on which the conclusions of their manuscript are based?**

We rewrote our results with the editors' comments.

4. **Is the manuscript presented in an intelligible form and written in standard English?**

We proceeded with a new and improved English translation of our manuscript.

5. **Reviewer #1:**
   5.1. **Abstract** :

The methods in the abstract were more specifically worded, the search terms, time and GRADE were added, and the GRADE had a more adequate description. In addition, the wording was corrected with respect to the comparison groups (Pages 2-3).

5.2. **Introduction**:

The wording of the Kitley et al study and the paragraph regarding AQP-4 was corrected. Further expanded the relationship of NLR as a marker in other diseases.(Pages 3-4).

5.3. **Methods**:

- Added search terms to the methods section.(Page 5).

- Detailed that there were no restrictions in the search, regarding time or language (Page 5).

- It was detailed that this work had a "peer review", referring to the fact that the analysis and selection of studies were made by 2 authors. (Page 6)

5.4. **Results:**

- It should be noted that our measure of effect is the difference in means, so the point of no effect is zero.

- The section on the extraction of the NLR values of the comparator was improved (Pages 5-7).

- We improved the wording to explain the definition of severe and mild NMOSD, as well as the use of EDSS criteria (Page 6).

- Improved wording was made on the follow-up of crude and adjusted association measures (Page 7).

- Improved wording on standard deviation extraction (Page 8).

- Improved statistical approach regarding random effects models and the use of the mean NLR difference as the outcome. (Page 8)

- Added NLR values in Table 1 for the NMOSD population as well as the controls. (Page 11-12)

- Detailed how the NLR was collected across all studies and the 95% CIs for each study. (Page 8)

6. **Reviewer #2**:
   6.1. Described in more detail the eligibility criteria as well as the pretreatment period (Page 5) 6.2.
   6.2. The fact that most of the studies were conducted in Chinese populations, which limits the overall transferability, was redacted in limitations (Page 19).
